# The fibroblast growth factor–Klotho axis at molecular level

**DOI:** 10.1515/biol-2022-0655

**Published:** 2023-10-27

**Authors:** Fuqiang Sun, Panpan Liang, Bo Wang, Wenbo Liu

**Affiliations:** School of Anesthesiology, Weifang Medical University, Shandong Provincial Medicine and Health Key Laboratory of Clinical Anesthesia, Weifang 261053, Shandong, China; School of Basic Medical Sciences, Air Force Medical University, Xi'an, 710032, Shaanxi, China; Central Laboratory of the First Affiliated Hospital, Weifang Medical University, Weifang 261000, Shandong, China

**Keywords:** Klotho, fibroblast growth factor, heparin, molecular mechanism

## Abstract

Klotho is a recently discovered protein that has positive effects on all systems of the body, for example, regulating calcium and phosphorus metabolism, protecting nerves, delaying aging and so on. Fibroblast growth factors (FGFs) are a group of polypeptides that function throughout the body by binding with cell surface FGF receptors (FGFRs). Endocrine FGFs require Klotho as a co-receptor for FGFRs. There is increasing evidence that Klotho participates in calcium and phosphorus regulation and metabolic regulation via the FGF–Klotho axis. Moreover, soluble Klotho can function as a separate hormone to regulate homeostasis on various ion channels and carrier channels on the cell surface. This review mainly explains the molecular basis of the membrane signaling mechanism of Klotho.

## 1 Introduction

The Klotho protein was discovered in 1997, and its name is derived from Clotho, the goddess of fate who determines the length of life, as per Greek mythology. Klotho protein was first found in the distal convoluted tubules of transgenic mice [1–4]. There are three types of Klotho in the human body, namely α-Klotho (KLA), β-Klotho (KLB), and γ-Klotho. Moreover, Klotho can also be divided into the membrane-binding type (mKL), secretory or soluble type (sKL), and intracellular type. These different forms of klotho are involved in different physiological processes [5]. The KLA gene is located on chromosome 13q12; it has five exons and four introns and encodes a type I single transmembrane protein (mKLA) with a molecular weight of 135 kDa which comprises 1,012 amino acids [6,7]. mKLA is mainly found in the kidney and comprises the extracellular domain (KL1 and KL2), N-terminal signal, ten amino acids at the C-terminal of the transmembrane helix, and the intracellular cytoplasmic domain [8]. mKLA is cleaved by a disintegrin and metalloproteinase 10/17(ADAM10/17) at the proximal end of the cell surface (α-cutting) and forms an isomer called the secretory-type KLA (sKLA), which has a molecular weight of 130 kDa. sKLA only contains KL1 and KL2 domains and is predominantly obtained by the hydrolysis of mKLA on the cell membrane of distal renal tubules. Therefore, the expression of sKLA is the most abundant in distal renal tubules. β-cleavage can also occur between the KL1 and KL2 domains of sKLA to form a 60 kDa isomer containing only the KL1 domain (intracellular type). Besides, the 3C end of the exon of the Klotho gene carries alternative splicing sites, and the translation of alternative mRNA splicing sites may lead to premature termination of codon translation and the formation of an inactive protein comprising KL1. Except for sKLA, all isomers of Klotho have limited blood circulation. The KLB gene is located on chromosome 4. Compared to KLA, KLB is only membrane-bound and has no secretory form; it is mainly found in the liver and white adipose tissue [8,9]. γ-Klotho is predominantly expressed in the kidney and skin; however, its function remains unclear. Thus, sKL in the human body is mainly sKLA. The sKL mentioned below is sKLA. Klotho expression is affected by many physiological and pathological conditions. The levels of Klotho are reportedly significantly reduced in the animal and human brain, kidney, atrial node, liver, and serum [10–18]. In addition, oxidative stress, inflammation, angiotensin II, aldosterone, and proteinuria inhibit Klotho expression [19]. This protein expression is also decreased in many diseases, such as Alzheimer’s disease [20–22], acute and chronic kidney disease [22,23], chronic obstructive pulmonary disease [24], diabetes mellitus (bamboo medium) [25–27], some cancers, and various vascular cancer pathologies, including arteriosclerosis, atherosclerosis, and stroke [28,29].

The fibroblast growth factor (FGF) family comprises 22 polypeptides that play important roles in embryonic development and normal tissue homeostasis by binding with FGF receptors (FGFRs) [30]. Endocrine FGFs, including FGF19, 21, and 23, are powerful endocrine hormones that regulate various aspects of physiological homeostasis [31]. The binding of endocrine FGFs and FGFRs requires KLA or KLB as a co-receptor [32]. FGF19 is a satiety hormone secreted in the intestine during food intake, which in combination with the KLB–FGFR4 complex of hepatocytes promotes the metabolic response of food intake [33]. Conversely, under fasting conditions, FGF21 is secreted by the liver; it binds to the KLB–FGFR complex of adipocytes and the suprachiasmatic nucleus to activate the hypothalamus–pituitary–adrenal axis and the sympathetic nervous system, consequently regulating the metabolic response to fasting and stress [34–38]. FGF23 is secreted by osteoblasts in response to phosphate uptake; it binds with the KLA–FGFR complex to regulate mineral metabolism [39–42].

## 2 Molecular structure of KLA/KLB

The KLA domains KL1 and KL2 comprise an inner eight-stranded parallel α-barrel and eight surrounding β-helices. The two domains of KLA are connected by a proline-rich rigid chain, N-terminal of β1chain, α7 helix of KL1, β5α5 rings, β6α6 rings, and α7 helix of KL2 [43]; furthermore, a special inter-domain contact is mediated by zinc ions, which promotes the activity of the co-receptor KLA–FGFR by minimizing the flexibility between domains. KLB is structurally similar to KLA. KL1 and KL2 domains of KLB comprise eight units each of β-slice and α7 helix; the inter-action domain of KLB has a wide network of hydrophobicity and polar interactions. KL1 and KL2 domains of both KLA and KLB are homologous with glucosidase-1 (GH1). GH1 contains two highly conserved glutamate residues, which are necessary for glucosidase to function. The first glutamate is a nucleophilic residue, and the second glutamate has the activity of acid–base catalysis double substitution mechanism [44–46]. GH1 hydrolyzes carbohydrates through a double substitution mechanism mediated by the two conservative glutamate residues. However, Asn241 replaces the first glutamate in the KL1 domain of KLB, whereas in the KL2 domain, Ala889 replaces the second glutamate [47], indicating that the glycoside hydrolase-like domains (KL1 and KL2) of KLB are not real glycoside hydrolases. KL1 and KL2 domains of KLA also have no glycoside hydrolase activity, possibly due to the replacement of the two conservative glutamates.

## 3 Molecular structure of FGF23–KLA–FGFR

### 3.1 Structural association between KLA and FGFR

**Figure 1:**
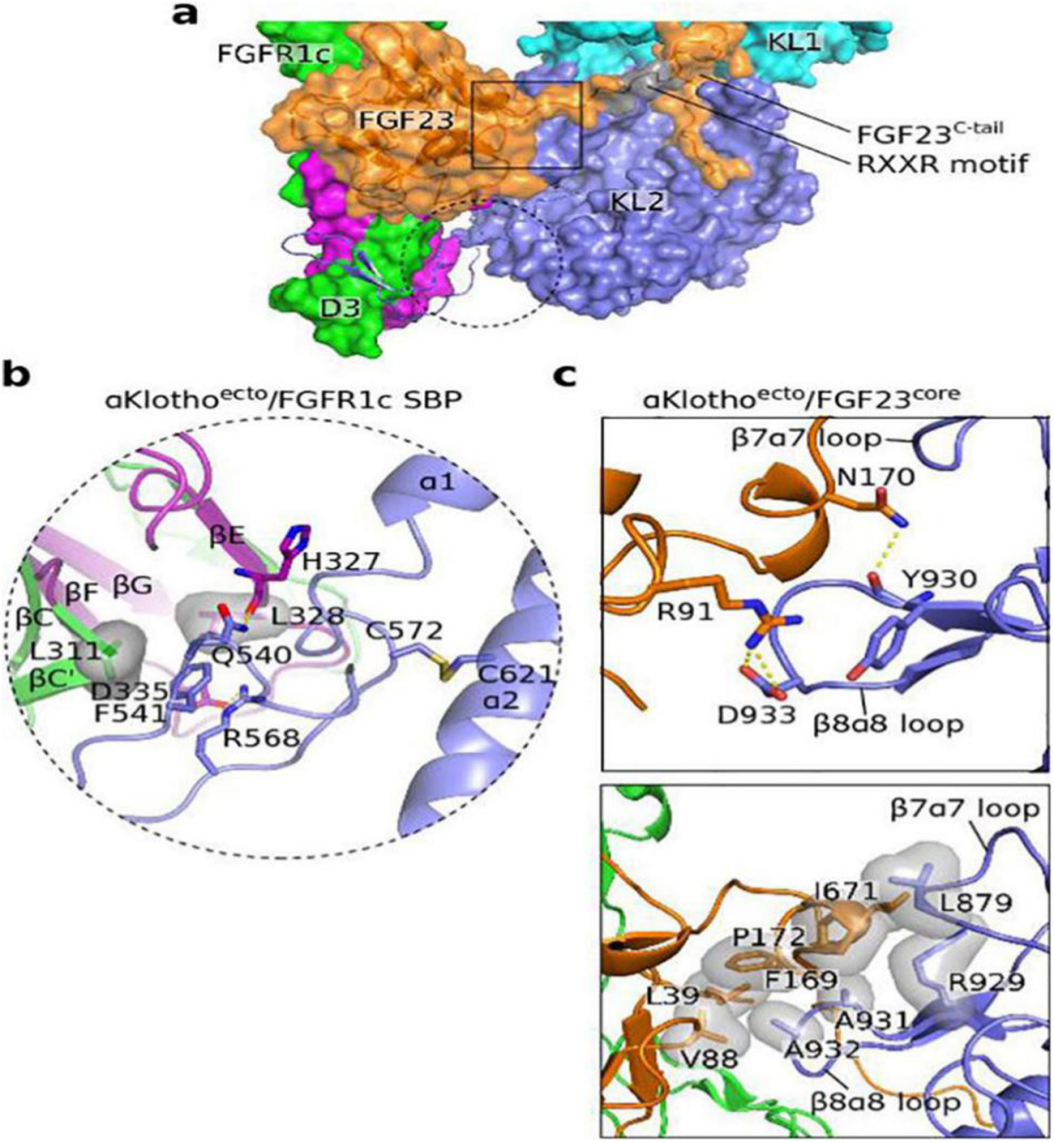
(a) View of the ternary complex FGF23–KLA–FGFR. The circular frame shows the binding of the RBA of Klotho with D3 domain of FGFR. The square frame shows the binding of FGF23CT with KLA. (b) Close-up view of the circular frame of panel (a). (c) Close-up view of the square frame of panel (a). Yellow surface represents hydrogen bonding and the gray translucent surface represents hydrophobicity. This image has been reproduced with permission from Chen et al. [43].

The individual entities of the ternary complex FGF23–KLA–FGFR have close interaction with each other, and the molecular conformation is shown in Figure 1a. FGFR comprises an extracellular ligand-binding domain, a transmembrane helix domain, and a cytoplasmic part with tyrosine kinase activity. The extracellular ligand-binding domain comprises three immunoglobulin-like domains (D1–D3). KLA is bound to FGFR primarily through the binding of the KL2 and D3 domains. The long β1α1 loop of KL2, which is a 35 kDa amino acid sequence extending from the KL2 nucleus, is locked in the FGFR D3 domain; it is called the receptor-binding arm (RBA). A short β-chain pair (RBA-β1: RBA-β2) is formed by the binding of distal residues of RBA (547Tyr–Leu–Trp549 and 556Ile–Leu–Arg558) and the FGFR D3 domain. RBA-β1: RBA-β2, the βC′–βC–βF–βG slice, and broad hydrophobic channels between the βC–βC′ rings of the FGFR D3 domain together form a large hydrophobic surface. In addition, the binding of RBA-β1 and βC of the FGFR D3 domain forms three hydrogen bonds to further strengthen the interface (Figure 1b). Although the RBA proximal residue is bound to a second smaller binding pocket at the bottom of D3, the disulfide bond between Cys-572 of the RBA N-terminal and Cys-621 of the KL2 α2 helix endows the interface with a certain degree of conformational rigidity, thus making the interface more stable [43,48].

### 3.2 Structural association between KLA and FGF23CT

FGF23CT is noted to have two KLA-binding sites, one of which is a KL-binding peptide, namely FGF23^180–205^ (C26, also known as the first KL site), and the other is a potential KL interaction site, namely FGF23^212–239^ (C28, also known as the second KL site); C26 and C28 have only 40% homology, and both contain DPL motifs; thus, they may interact differently with KLA [49,50]. In this article, we mainly introduce the interaction between KLA and the first KL site of FGF23. In the interface between KLA and FGF23CT, the DPL motif of FGF23 residues (^188^Asp–Pro–Leu–Asn–Val–Leu^193^) is the most important and is bound to residues between KL1 and KL2 through an unusual “cage” structure to form hydrogen bonds and hydrophobic bonds. Notably, Tyr-433 in the KL1 α7 helix plays an important role in fastening the “cage” structure of FGF23CT, which is required for accurate alignment of KL1 and KL2 residues. Zn^2+^ is the auxiliary group of KLA and promotes accurate alignment of residues by minimizing the flexibility between KL1 and KL2 domains (Figure 1c). In the downstream FGF23CT, the side chains of basic amino acids (Lys-194, Arg-196, and Arg-198) combine with the residues in the center of KL2 to form multiple hydrogen bonds. At the interface between the β-trefoil nucleus and KLA, the α-C helix of FGF23 is bound to short β7–ɑ7 rings and β8–ɑ8 rings in the upper margin of KL2 cavity to form hydrogen bonds and hydrophobic bonds. The combination of the three points mentioned above improves the stability of FGF23–KLA [43,48].

### 3.3 Structural association between FGFR and FGF23

The N-terminal of FGF23 is mainly bound to D2 and D3 domains of FGFR, albeit weakly, and the interaction between D2 and D3 domains is also weak, which results in low intrinsic affinity of FGF23 and FGFR. Therefore, FGF23 is equivalent to a linker of D2 and D3 domains. KLA is a non-enzyme scaffold and combines with FGF23 and FGFR, which makes FGF23 and FGFR adjacent to each other, thus allowing an increased affinity between them [43]. Currently, the molecular mechanism of interaction between FGF23 and FGFRs remains unclear.

There are two forms of KLA: mKLA and sKLA. However, FGF23 mainly interacts with mKLA and not so prominently with sKLA because sKLA contains only KL1 and KL2 domains and does not contain the AA transmembrane structure or an intracellular short domain, thus weakening the interaction between FGF23 and sKLA. This may reduce the signal transduction efficiency of FGF23. Perhaps FGF23 and sKLA combine in a different way to play a unique physiological role; however, this remains to be elucidated.

The FGF23–KLA–FGFR complex plays an important role in maintaining a balanced state of phosphate metabolism in the body. First, it can reduce vitamin D biosynthesis and cellular reabsorption of phosphorus, accelerate urinary phosphorus excretion, and thereby reduce the incidence of vascular calcification [51]. Second, it can inhibit the expression of proteins related to calcium and phosphorus metabolism in the proximal tubules, play a role in inhibiting renal reabsorption of phosphorus, and regulate the overexpression of phosphorus [52]. Finally, besides acting on the kidneys to regulate phosphorus absorption, it can also prevent intestinal absorption of phosphorus by regulating the expression of the related proteins in intestinal cells and reducing the concentration of phosphorus in urine and blood in the body [53]. In summary, abnormal expression of FGF23–KLA–FGFR can severely affect the balance of calcium and phosphorus metabolism in the body (Figure 4).

## 4 Molecular structure of FGF21/FGF19–FGFR–KLB

### 4.1 FGF19CT and FGF21CT bind to KL1 domain of KLB through DPL motif

**Figure 2:**
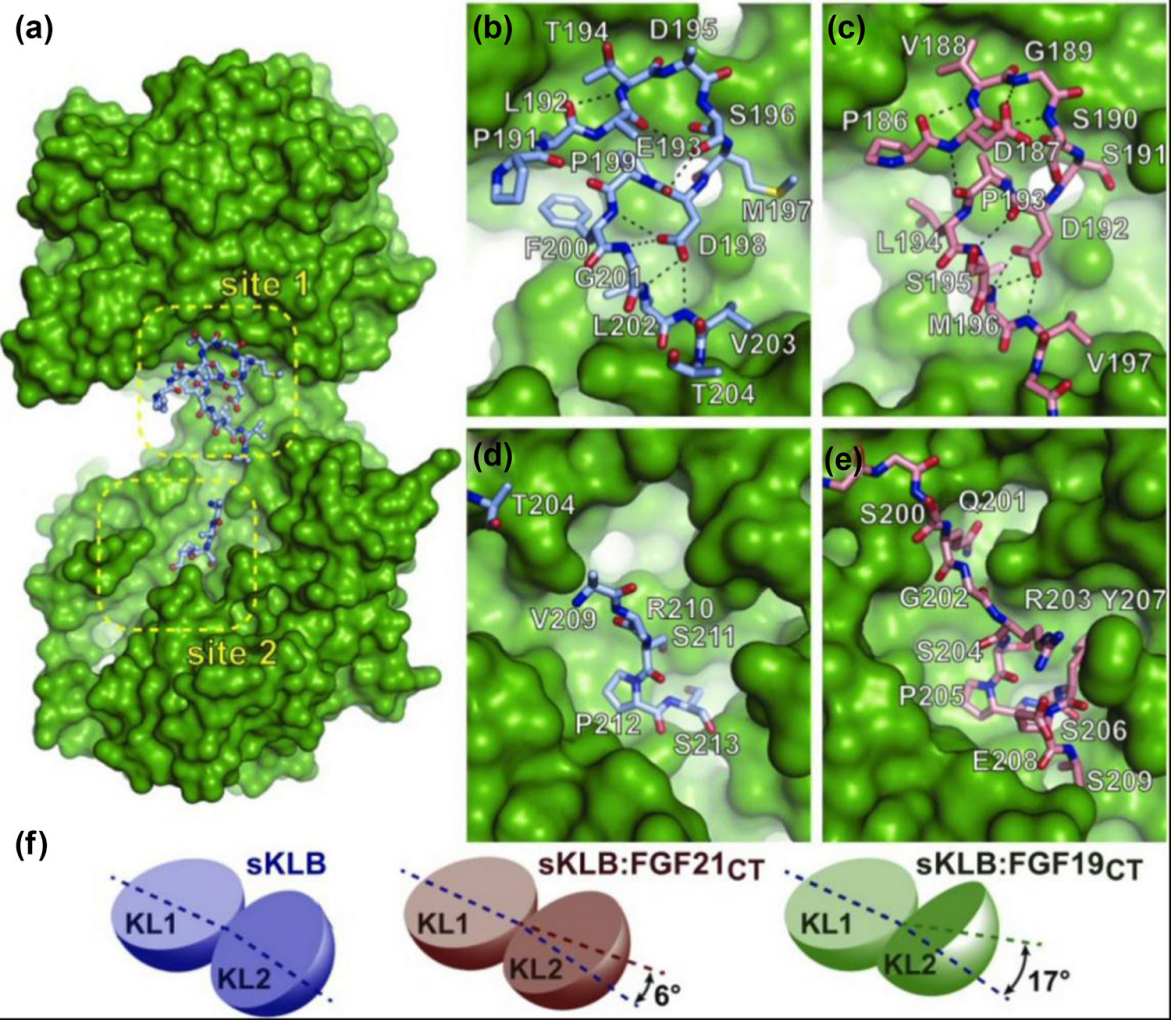
(a) There are two binding sites of KLB to FGF19/21 – sites 1 and 2. (b) Residue of FGF19 binds to the KLB site 1. (c) Residue of FGF21 binds to KLB site 1. (d) Residue of FGF19 binds to KLB site 2. (e) Residue of FGF21 binds to KLB site 2. (f) KLB binds to FGF19/21. The spatial structure of KL1 and KL2 changes. This image has been reproduced with permission from Kuzina et al. [54].

FGF21CT and FGF19CT bind to both the KL1 and KL2 domains of KLB. The binding sites of FGF19CT and FGF21CT in the KL1 and KL2 domains of KLB are called site 1 and site 2, respectively (Figure 2a). The KL1 domain of KLB could bind to the amino acid (^186^Pro–Val^197^) of FGF21CT via hydrophobic interaction. Two kinds of type I turns comprising ^187^Asp–Val–Gly–Ser^190^ and ^192^Asp–Pro–Seu–Ser^195^ and the ST-turn formed by ^190^Ser–Ser–Asp^192^ are combined to form the ligand region of FGF21CT, which could bind to the KL1 domain of KLB [47]. Meanwhile, the DPL motif (^192^Asp–Pro–Leu^194^) included in the type I turn may play an important role in the binding of the KL1 domain of KLB with the ligand (Figure 2c). Similarly, DPL motifs are noted to exist in the P191–V203 motif of FGF19CT, and they form a large hydrophobic surface with the KL1 domain of KLB [47,50,54] (Figure 2b). However, it is currently not clear whether the ligand region of FGF19CT, like FGF21CT, has the structural rigidity to promote stable binding.

### 4.2 FGF19CT and FGF21CT bind to the KL2 domain of KLB through the S–P–S motif

Half of the FGF21CT sequence (S–Q–G–R–S–P–S–Y–A–S) contains hydroxyl side chains and could bind to KL2 of KLB; therefore, this region of FGF21 appears to mimic the glucoside substrate [55,56]. This sequence also contains the S–P–S sequence (^204^Ser–Pro–Ser^206^), which is key for the binding of FGF21CT and the KL2 domain of KLB (Figure 2e). The hydroxyl groups of Ser204 and Ser206 in FGF21CT interact with the carboxyl group of Glu693 in KLB to simulate the reaction between GH1 and glucoside substrate. In the Koshland disubstitution reaction of GH1, Glu693 is one of the two conserved catalytic glutamic acids and acts as a general acid–base catalyst. Pro205 of FGF21CT combines with Phe826, Phe931, and Phe942 of KLB through hydrophobic interaction to further strengthen the interface [47,54]. The S–P–S motif (S211–P212–S213) of FGF19 binds to the KL2 domain of KLB (Figure 2d) [54]; however, the specific combination method remains unidentified. They all bind to the substrate-binding region of the KL2 domain through the S–P–S motif (similar to sugar sequence).

### 4.3 Binding of FGF19CT and FGF21CT to KLB was affected by the change of spatial conformation and electrostatic potential distribution

**Figure 3:**
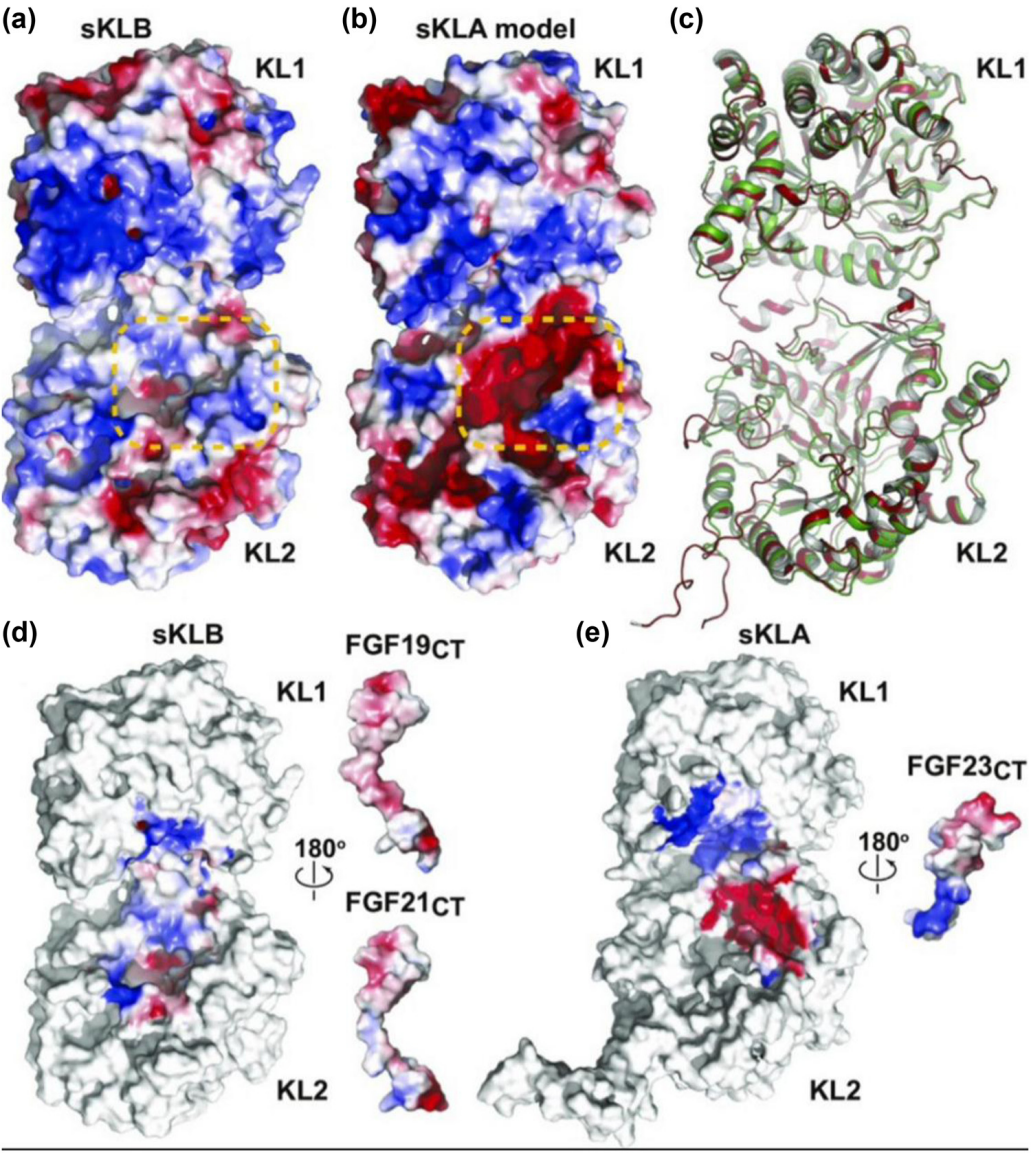
(a) Electrostatic potential distribution of sKLA and sKLB. Red is the negative potential and blue is the positive potential. Orange dotted box: the main difference of electrostatic potential distribution between them. (b) Comparison of the crystal structures of sKLA and sKLB, which almost overlap each other. (c) Comparison of crystal structures of sKLB and sKLA. (d) Relationship between sKLB and FGF19/21CT. (e) Electrostatic interaction between sKLA and FGF23CT. Reproduced with permission from Kuzina et al. [54].

**Figure 4:**
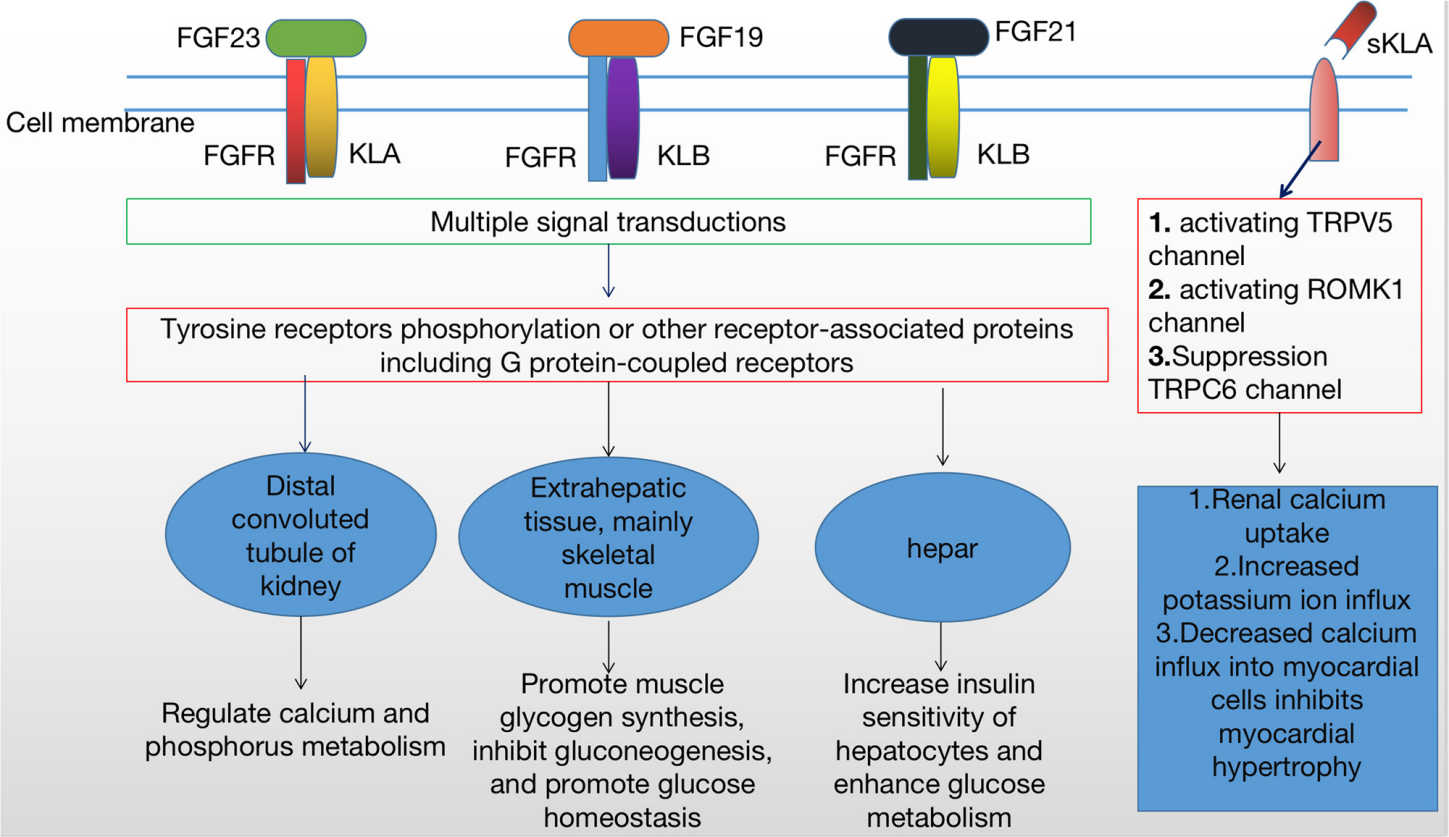
Schematic of the biological effects of FGF–Klotho axis and sKLA.

The combination of FGF21CT/FGF19CT with KLB requires KL1 and KL2 domains to interact with each other, which changes the distance and angle between KL1 and KL2 domains. The main features are as follows: the connection of KL1 and KL2 domains is flexible, the binding of FGF21CT with KLB results in the inter-domain angle of KL1 and KL2 rotating 6°, and the binding of FGF21CT with KLB results in the inter-domain angle of KL1 and KL2 rotating 17° (Figure 2f), which may affect the binding of FGF19CT/FGF21CT with KLB. In terms of electrostatic potential distributions, the electrostatic potential distribution is different for KLB and KLA, but their crystal structures almost overlap (Figure 3c), which may be attributed to the different structures of the KL2 domains of KLA and KLB. The tyrosine (Y809 and Y915) of KLA is the key amino acid responsible for the negative electrostatic potential. In the KL2 domain of KLB, tyrosine (Y809 and Y915) is replaced by phenylalanine (F826 and F931), resulting in positive electrostatic potential. The KL2 domain of KLA, which has negative electrostatic potential, is bound to FGF23CT, which shows positive electrostatic potential centered on R196 and R198 (Figure 3a and b); conversely, owing to the S–P–S motif, FGF23CT shows negative electrostatic potential and combines with the KL2 domain of KLB with positive electrostatic potential, which may at least partly explain why the KL2 domain of KLA does not bind to FGF23CT through the S–P–S motif. This also indirectly proves that the difference in the electrostatic potentials of KL2 domains is instrumental in determining the specificity of ligand binding. Due to the differences in amino acids on both sides of the S–P–S motif, FGF19CT shows a slightly stronger negative electrostatic potential than FGF21CT (Figure 3d and e). Therefore, the combination of FGF21CT and KLB is closer than that of FGF19CT and KLB [54,57]. KLB is the main receptor of FGF21, a hormone produced during starvation. FGF21 can combine with FGFR and KLB to increase insulin sensitivity, enhance glucose metabolism, reduce blood sugar, and induce weight loss. At the same time, KLB can also bind FGF19 and FGFR to activate extrahepatic tissues, mainly acting on skeletal muscles, promoting muscle glycogen synthesis, inhibiting gluconeogenesis, and promoting glucose homeostasis, and it may thus become a new treatment target for diabetes and obesity (Figure 4).

## 5 Connection and difference between FGF23 and FGF19/21

**Figure 5:**
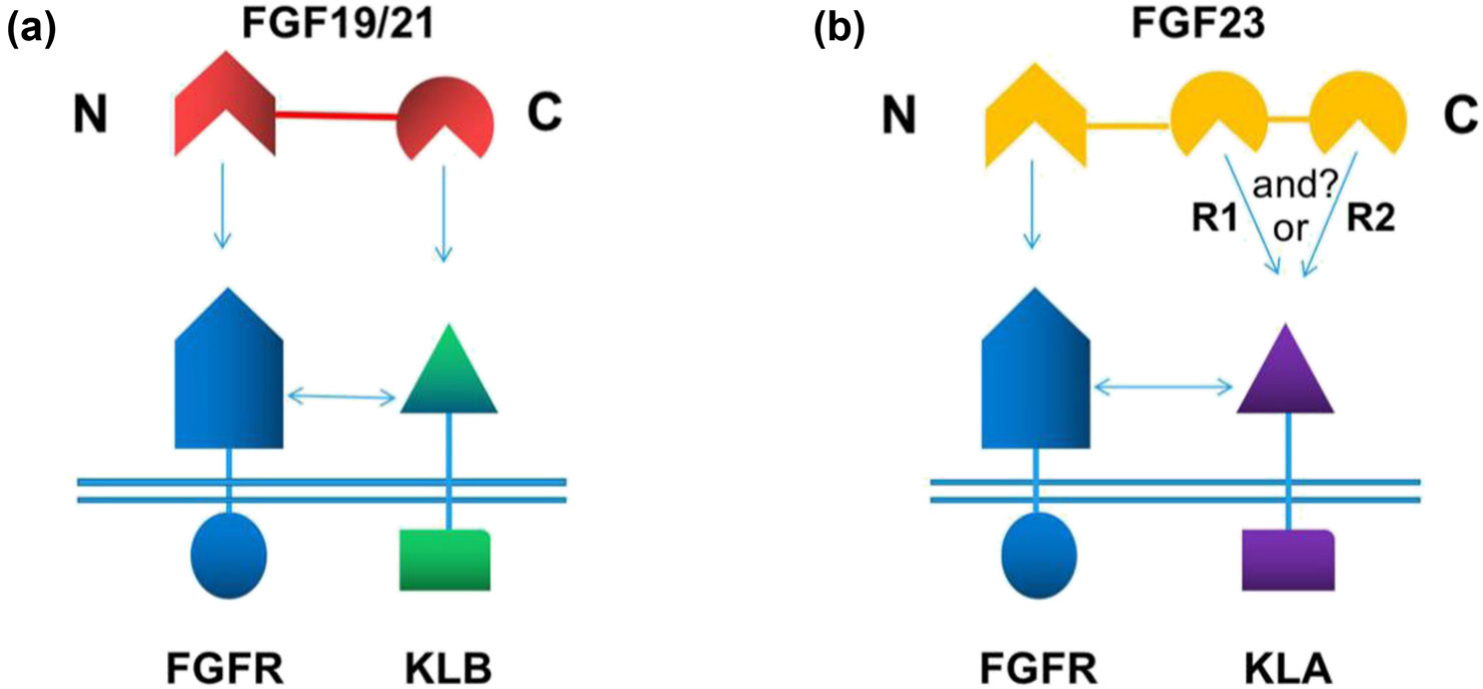
(a) Schematic diagram of the combination of FGF19/21 and KLB. (b) Schematic diagram of the combination of FGF23 and KLA. The “or” represents the combination of R1 and R2 with KLA, and “and” represents the combination of R1 and R2 with KLA; however, it is not yet clear whether this mechanism exists.

FGF23, FGF19, and FGF21 are bound to the KL1 domain of KLA and KLB through the DPL/F motif. The sugar mimetic pro-tease (S–P–S) motif of sucrose phosphate synthase in FGF19 and FGF21 is bound to the KL2 domain of KLA. FGF23CT does not contain the S–P–S sequence but is bound to the KL2 domain of KLB through some basic amino acids [43,47,58]. However, the biggest difference between FGF23 and FGF19/FGF21 is structural diversity. Previous studies have shown that FGF23CT has 89 amino acids and two tandem repeats (R1 and R2) with high affinity for KLA; therefore, the binding between FGF23 and KLA is called divalent binding. The C-terminal tail of FGF19 and FGF21 could only bind to one KLB (Figure 5a and b). Divalent FGF23 could stimulate dimerization between pre-existing KLA–FGFR and a free KLA molecule or another pair of pre-existing KLA–FGFR. Although the binding affinity of R1 and R2 to free KLA is very similar, KLA–FGFR may preferentially interact with R1, whereas the two cysteines connected by disulfide bonds on both sides of R2 may tend to bind with the free KLA molecule [50]. When one active site of FGF23 is lost, the other action site would compensate, and FGF23 would be inactivated only when two action sites are lost at the same time [49]. The formation of a dimer requires two FGF23, but due to the bivalent interactions of the two sites of FGF23, one FGF23 active site may satisfy the formation of a dimer (Figure 5b). In our opinion, the formation of a dimer would require the participation of both FGF23 binding sites because the spatial structure of R1 and R2 of FGF23CT is almost identical, and R1 of FGF23 has high affinity to KLA–FGFR. Nevertheless, these problems still warrant further study.

## 6 Formation of 2:2:2:2 FGF–FGFR–Klotho–heparin sodium (HS) dimer

HS is essential for FGFR dimerization, activation, and cell proliferation [32,59,60]. Considering the classic FGF as an example, in the 2:2:2 FGF–FGFR–HS model, HS is closely bound to 1:1 FGF–FGFR and interacts with the D2 domain of FGFR in the adjacent 1:1 FGF–FGFR. HS is bound to the dimer by 30 hydrogen bonds, 25 of which are between the 1:1 FGF–FGFR complex and HS, and the remaining 5 are formed between the adjacent 1:1 FGF–FGFR complex and HS.

**Figure 6:**
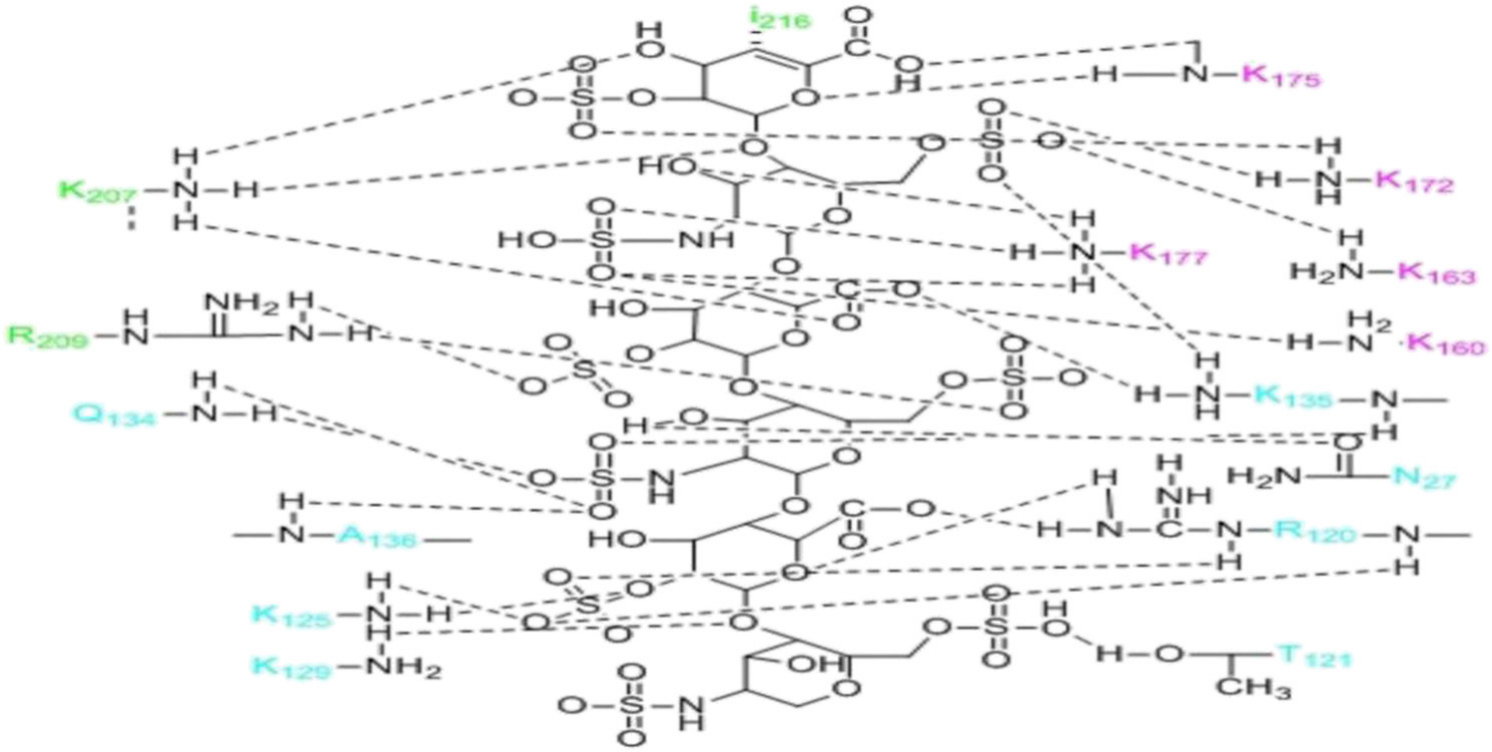
FGF, FGFRD2 domains, adjacent FGFR2 domains, and other amino acids interact with HS. Blue is the residue of FGF, green is the residue of the adjacent FGFRD2 domain, and pink is the residue of the FGFD2 domain. There are six rings from top to bottom, which are A, B, C, D, E, and F rings.

Notably, FGF is bound to HS by 16 hydrogen bonds, 10 of which are mediated by sulfate and 6 by heparin carboxylate, linker, and epoxide. FGF surface residues (e.g., Asn-27, Arg-120, Thr-121, Lys-125, Lys129, Gln-134, Lys-135, and Ala-136) constitute heparin-binding sites [61,62]. The binding of the FGFR D2 domain with HS involves nine hydrogen bonds, which are mediated by heparin N-sulfate, 2-O-sulfate, and 6-O-sulfate. At the interface between HS and the adjacent 1:1 FGF–FGFR, the A–D ring of HS is only bound to the amino acids in the D2 domain of FGFR (e.g., Lys-207, Arg-209, and Il-216) to form five hydrogen bonds. The hydrogen bond between Lys-207 and HS is mediated by heparin carboxylate, linker, and epoxide. Arg-209 forms hydrogen bonds with the 2-O-sulfate group of ring C and the 6-O-sulfate group of ring D. Hydrophobic contact between Il-216 and ring A further enhances the interaction (Figure 6) [62,63]. However, in the above process, only FGF23 from the members of the FGF19 subfamily has been confirmed to require the involvement of HS [64]. Whether HS participates in the formation of FGF19/21 signal complexes remains to be studied. Although the possibility of FGF19/21 binding to HS is very low, there may be other molecules besides HS involved in the formation of signal complexes.

**Figure 7:**
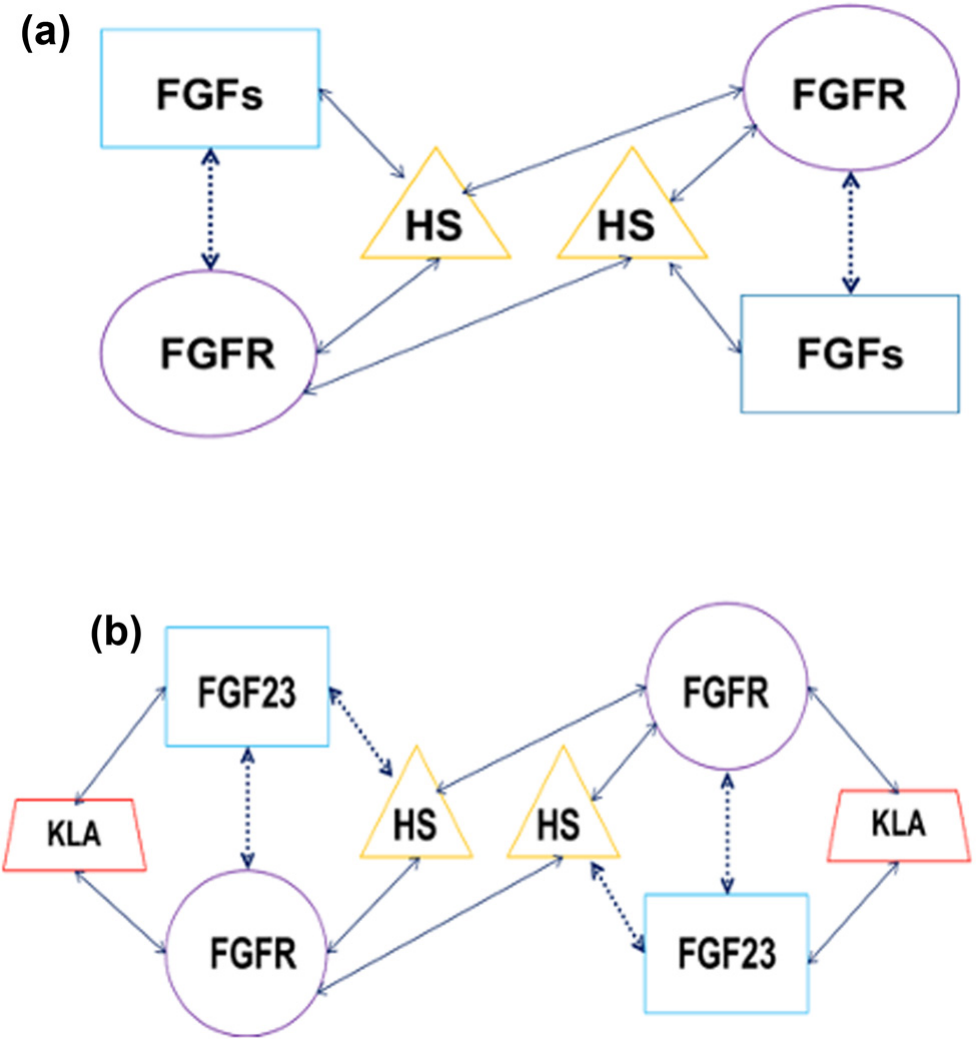
(a) Classical FGF, HS, and FGFR form a ternary complex and then form a dimer. (b) Endocrine FGF-19 subfamily and HS, Klotho, and FGFR form a ternary complex and then form a dimer. Solid lines represent a close relationship between substances, and dotted lines represent a weak relationship between substances.

The HS-binding region of the FGFR D2 domain is highly conserved, whereas the HS binding of FGF shows considerable diversity [62,65,66]. There is a large distance between the 1–2 ring and 10–12 ring of the FGF19 subfamily that lacks the GXXXXGXX (T/S) motif, which results in greatly reduced binding ability of FGF19 subfamily members with HS. The nuclear homologous region of classical FGF is folded into 12 antiparallel chains (β1–β12) to form a spherical area called Trifolium. All members of the FGF19 subfamily lack β11 chains containing the T/S motif. Although the residues Lys149 to Lys155 of FGF19 form an α11 spiral to replace β11 chains and FGF23 uses g11 helix to replace β11 chains, the affinity of FGF19 subfamily to HS is still very low [67]. Therefore, HS is not enough to promote the formation of 1:1 FGF–FGFR, at this time, some assistance from Klotho is needed. The affinity between HS and FGFR remains unchanged, which plays a role in the dimerization of two adjacent ternary complexes (FGF19s–Klotho–FGFR) (Figure 7).

## 7 Functions of sKLA are independent of FGF–FGFR forms

As mentioned above, ternary complexes are formed by mKLA, FGF23, and FGFR, and the two ternary complexes form a dimer in the presence of heparin. The tyrosine kinase in FGFR is phosphorylated, and the FGFR substrate 2 and the downstream targets ERK1 and ERK2 are phosphorylated as well, thus resulting in corresponding physiological effects. KLB only has a membrane-bound type, and therefore, the sKL mentioned here is sKLA. sKLA, a circulating anti-aging hormone, functions independently of FGF23 and is considered to lack co-receptor activity. Although sKLA could form complexes with FGF23 and FGFR, the signal transduction level of sKLA is far lower than that of mKLA. Furthermore, the physiological concentration of sKLA is too low to meet the formation of FGF23 co-receptor. Therefore, we speculate that the physiological function of sKLA may not be related to FGF23 [68,69]. Studies have found that sKLA and heparin mediate the binding of FGF23 with different types of FGFRs. Heparin specifically mediates FGF23 binding to FGFR4, whereas sKLA mediates FGF23 binding to other types of FGFRs. The specific type of FGFR needs to be studied. sKLA and HS have the opposite effect in regulating myocardial hypertrophy. In other words, decreased sKLA and increased heparin can induce myocardial hypertrophy. Therefore, sKLA is also involved in the formation of the FGF23–FGFR complex under certain conditions [70]. KL1 and KL2 domains of sKLA are homologous to mammalian lactose hydrolase 1 (GH1) but lack two conserved glutamate residues with acid–base catalysis [46,71]; therefore, sKLA may not have real glycosidase activity but worked as a lectin instead.

sKL regulates the activities of various ion channels and transporters, including transient receptor potential cation channel V5 (TRPV5) and the renal outer medullary potassium (ROMK1) channel. The membrane receptor of sKLA is a ganglioside containing α-2,3-sialic lactulose. sKLA exerts pseudoglucosidase activity and hydrolyzes the glycosylated chain of α-2,3-sialiclactulose on the cell surface [72,73], which exposes the disaccharide *N*-acetolactate amine (LacNAc). The binding of calectin-1 with LacNAc causes the accumulation of functional changes of plasma membrane channels, which results in increased calcium absorption and potassium secretion [74]. However, recent studies have suggested that sKLA is a lectin and not an enzyme. sKLA, which has the galectin1 ligand, perhaps binds to the α-2,3-sialic lactulose of channel protein indirectly through galectin1, which increases the abundance of TRPV5 and ROMK1 [26,75]. sKLA promotes the function of most transporters and channel proteins; however, there are exceptions, such as the presence of transient receptor potential cation channel 6 (TRPC6), long-term high pressure, abnormal calcium signal, activated calcineurin and the nuclear factor of activated T cell, upregulated expression of the TRPC6 gene, and increased TRPC channel protein on the cell surface, which lead to increased calcium influx and long-term enhancement of myocardial contractility, eventually causing cardiac hypertrophy [26,75]. sKLA is bound to TRPC6 through the action of pseudoglucosidase and inhibits the function of TRPC6, and simultaneously, it inhibits the binding of IGF-1 to its receptor, inhibits P13K, and blocks the exocytosis of TRPC6. Therefore, sKLA could inhibit the quantity and function of the TRPC6 channel protein and may play a therapeutic role in hypertrophic heart disease induced by various stressors [76–78] (Figure 4).

## 8 Conclusion

**Figure 8:**
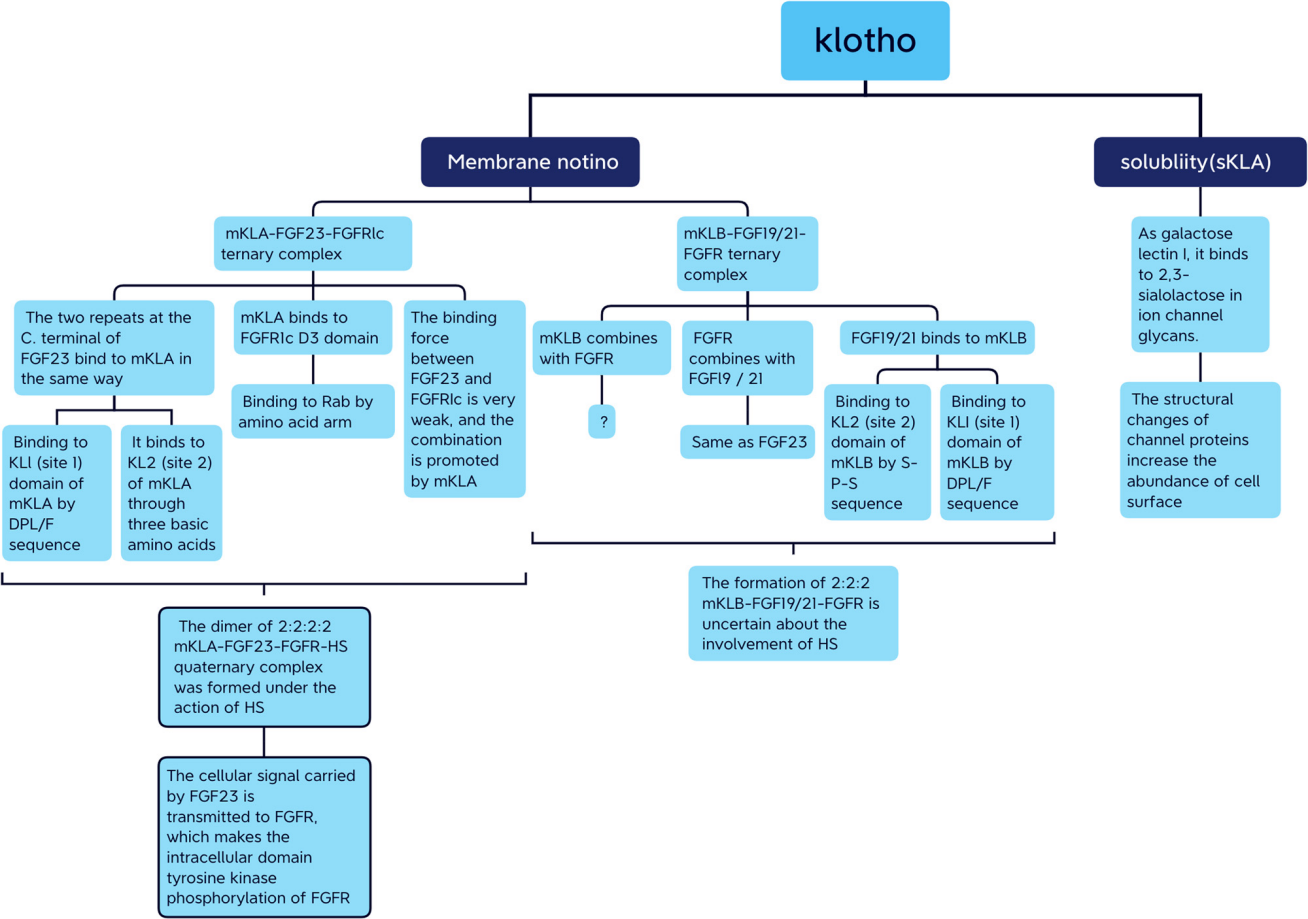
Flow chart summarizes two types of Klotho which play corresponding physiological roles in their respective modes.

mKL is a co-receptor of endocrine FGFs and has a role similar to that of HS in the signal transduction of classical FGFs. The binding affinity of endocrine FGFs and Klotho is 1,000–10,000 times higher than that of endocrine FGFs and FGFRs. Therefore, Klotho is the main surface receptor of endocrine FGFs, and FGFRs are the catalytic subunit of the activated signal complexes. However, the acid–base catalytic principle of FGFR and the role of acid–base catalysis in the process of signal transduction remain unclear. sKL breaks away from this mode of action and uses its own KL1 and KL2 domain pseudoglycosidase activities to bind to the receptor on the cell surface via enzyme–substrate binding, thus activating the cell physiological response. sKL is widely distributed and transported to various organs and tissues through blood circulation, leading to important physiological effects with anti-aging, anti-inflammatory, and anti-oxidative properties (Figure 8). Although many hypotheses have been put forward to explain the interaction of the FGF–Klotho–FGFR complex, the crystal structure of the FGF–Klotho–FGFR complex still needs to be explored to better understand their interaction, which will help elucidate the role of Klotho in the FGF–Klotho axis.
